# Arrested-motility states in populations of shape-anisotropic active Janus particles

**DOI:** 10.1126/sciadv.abo3604

**Published:** 2022-07-01

**Authors:** Jaideep Katuri, Ruben Poehnl, Andrey Sokolov, William Uspal, Alexey Snezhko

**Affiliations:** 1 Argonne National Laboratory, 9700 S. Cass Avenue, Lemont, IL 60439, USA.; 2 Department of Mechanical Engineering, University of Hawai’i at Mānoa, 2540 Dole Street, Holmes Hall 302, Honolulu, HI 96822, USA.

## Abstract

The emergence of large-scale collective phenomena from simple interactions between individual units is a hallmark of active matter systems. Active colloids with alignment-dominated interparticle interactions tend to develop orientational order and form motile coherent states, such as flocks and swarms. Alternatively, a combination of self-propulsion and excluded-volume interactions results in self-trapping and active phase separation into dense clusters. Here, we reveal unconventional arrested-motility states in ensembles of active discoidal particles powered by induced-charge electrophoresis. Combining experiments and computational modeling, we demonstrate that the shape asymmetry of the particles promotes the hydrodynamically assisted formation of active particles’ bound states in a certain range of excitation parameters, ultimately leading to a spontaneous collective state with arrested motility. Unlike the jammed clusters obtained through self-trapping, the arrested-motility phase remains sparse, dynamic, and reconfigurable. The demonstrated mechanism of phase separation seeded by bound state formation in ensembles of oblate active particles is generic and should be applicable to other active colloidal systems.

## Teaser

Shape asymmetry of active particles promotes hydrodynamically assisted bound states in a certain range of excitation parameters.

## INTRODUCTION

Active matter systems are composed of individual units that transduce energy from their local surroundings into self-propulsion or exert mechanical force (*1*). The sustained energy uptake and dissipation at the scale of the active unit drive these systems out of equilibrium, and they exhibit a number of complex phenomena that are inaccessible at thermal equilibrium. Active systems demonstrate a strong propensity toward the onset of large-scale collective behavior emerging from simple local interactions between active agents. In living systems, coherent collective states can be observed at all length scales, from the subcellular range of the cytoskeleton (*2*), to the micrometer scale of bacterial swarms and vortices (*3*), and the more familiar examples of bird flocks (*4*) and fish schools (*5*).

Over the past decade, there has been a substantial effort to develop synthetic active systems based on simple colloidal particles (*6*–*9*). The artificial systems share many properties with their biological counterparts, both at an individual level (*10*–*13*) and at a collective scale (*14*–*23*). Broadly, two types of collective behavior are observed in these systems, depending on the type of dominant interparticle interaction. In systems where velocity-alignment interaction is dominant, at sufficiently low fluctuations and high density, the system transitions from isotropic gas to spatially localized collective swarms that swim in a gas-like sea of particles (*14*, *15*). Upon further increase of particle density, the system transitions into a homogeneous polar liquid phase that spontaneously flows along a certain direction. When spatially confined, these systems have also been shown to form globally correlated vortices (*18*, *19*). Where macroscopically ordered motile states stem from alignment, the interparticle interactions have been shown to be dominated by hydrodynamics and electric or magnetic interactions (*14*, *15*). The second class of collective behavior is where the system phase separates into a dense cluster of low-motility particles in a sea of free-moving particles, at sufficiently high particle velocities and densities (*22*). Intriguingly, this transition can occur in the absence of any attractive particle interactions that would be necessary in a passive system. The formation of clusters arises solely from a combination of self-propulsion and isotropic excluded volume interactions that dominate over alignment interactions. This transition is an outcome of positive feedback between accumulation-induced slowing and slowing-induced accumulation of active particles and can be explained via a self-trapping mechanism, a phenomenon known as motility-induced phase separation (MIPS) (*24*).

Because MIPS is only dependent on particle velocity and density, there is little external control over the observed collective phase. This is unlike alignment-dominated systems, where the collective phase can be readily reconfigured through the parameters of the external field, because it is often dependent on interparticle hydrodynamic and field interactions. This control has been demonstrated in Quincke rollers (*19*, *25*), magnetic rollers (*14*), and electrokinetic particles (*26*) where the system can be switched from one collective state to another. Here, we demonstrate a system of synthetic discoidal self-propelled particles that spontaneously form aggregates of reduced motility, an arrested-motility phase, driven by the particles’ response to hydrodynamic fields created by their neighbors. The arrested-motility phase occurs spontaneously as a result of a positive feedback loop between slowing down of particles through hydrodynamics-induced pair formation and subsequent hydrodynamic trapping of single particles by the pairs, which seeds further pair formation. Unlike the clusters in MIPS, the arrested-motility phase is composed of groups of particles that are separated by a finite distance, and its properties such as spatial density and reversibility can be controlled by the driving field.

## RESULTS

### Experimental system

**Fig. 1.**
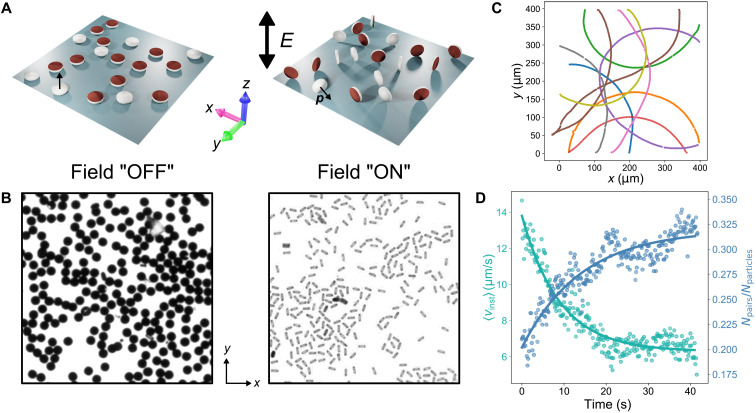
Shape-anisotropic active Janus particles. (**A**) Schematic of disk-shaped particles made of SU-8 (white) with axisymmetric coverage by a metal layer (brown) placed between two conductive indium tin oxide (ITO) glass slides (gray, only bottom glass shown). In the absence of an AC field, the particles settle face down on the glass slide. When an AC field in applied, the Janus particles orientation (normal to the disk) becomes perpendicular to the direction of the applied field. (**B**) Microscopy image of disk-shaped Janus particles (10 μm in diameter) on a transparent ITO glass slide. The particles appear as dark circles. The image contrast is enhanced for a better visualization. When an AC voltage is applied (5 V_pp_, 500 Hz), the particles stand up and appear as thin rods under the optical microscope. (**C**) Representative trajectories of disk-shaped active particles upon activation of the AC voltage at low particle density. (**D**) Time evolution of average ensemble velocity (cyan) and number of particle pairs (blue) in the system.

In our experiments and simulations, we investigate an active system of discoidal metallo-dielectric Janus particles (10 μm in diameter) energized by an external homogeneous alternating (AC) electric field. These particles are self-propelled by a mechanism known as induced-charge electrophoresis (ICEP) (*27*). When a low-frequency (100 Hz to 10 kHz) AC electric field is applied, the induced polarization of the metal and the dielectric sides are unequal because of the difference in their polarizability, which sets up ionic flows of different magnitudes on each side of the particle and results in self-propulsion toward the dielectric side. At zero electric field, particles lie flat with the orientation vector **p** perpendicular to the bottom surface with either the metal or the dielectric side facing up (Fig. 1, A and B, left). When an alternating electric field, *E* > 50 mV/μm is applied, it instantly causes the disks to stand up with the orientation vector becoming parallel to the surface, and they appear as thin rods under the microscope (Fig. 1, A and B, right). The particles maintain this orientation as long as an electric field of sufficient magnitude is applied and self-propel toward their dielectric side with a constant velocity *v*_p_.

In a dilute suspension of Janus disks, this system is highly motile (Fig. 1C) and the particle trajectories tend to be circular, reminiscent of bacterial trajectories close to a surface. In contrast with bacterial motion, the curvature of a disk trajectory originates from minor imperfections on the particle surface; see the Supplementary Materials for the in-depth characterization of individual behavior of disk-shaped active particles. We find that while the propulsion velocity is dependent on the magnitude of the applied electric field *E*, the curvature of the trajectories is controlled by the electric field frequency *f* (see fig. S1). This allows us to independently control both translational and the rotational behavior of the active particles directly by the parameters of the driving field.

**Fig. 2.**
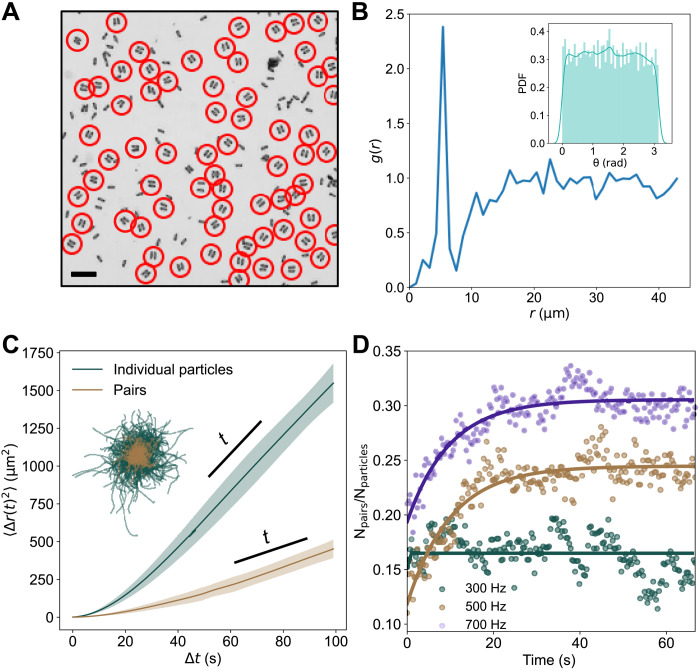
Pair formation in disk-shaped active Janus particles. (**A**) Microscopy image showing pair formation in systems of disk-shaped active Janus colloids. Pairs are identified by red circles. Scale bar, 40 μm. (**B**) The radial distribution function of particles *g*(*r*) in the arrested-motility phase. *E* = 0.15 V/μm and *f* = 500 Hz. Inset is the histogram of the particle pair orientation angles during the experiment. (**C**) Mean squared displacement curves obtained from the trajectories of pairs and individual particles. Dotted line is linear fit and shaded error region represents SD. Inset: Comparison of the trajectories of individual particles (green) and pairs (gold) centered at (*x*, *y*) = (0, 0). (**D**) Time evolution of the fraction of pairs to particles in systems of disk-shaped active Janus particles at different frequencies.

At surface area fraction Φ_s_ > 10%, collective behavior of the Janus discoidal particles begins to emerge. Within a few seconds of activation, the motility of the particle system drops substantially, and particle trajectories become strongly confined (see movie S1). The average ensemble particle velocity 〈*v*_inst_〉 reduces from 12 to 14 μm/s at the time of activation to 6 to 7 μm/s within a few seconds (Fig. 1D). Simultaneously, the observed motility-arrested state leads to the formation of particle clusters. Initial observation suggests that the arrested-motility phase is composed primarily of particles in a paired configuration (Fig. 1D). Unlike the dense areas formed by MIPS, we find that particles in these clusters are not jammed but rather spaced at a distance of several micrometers from each other (see Fig. 2A).

To determine whether there exists a characteristic interparticle separation distance in the arrested-motility phase composed of the particle pairs (Fig. 2A), we plot the radial distribution function *g*(*r*) for the particles (Fig. 2B). A sharp peak at ≈ 7 μm indicates that the particles prefer a certain spacing between each other at short distances. Closer observation of the low-motility phase shows that the system at high densities is primarily composed of stable bound particle pairs separated by a liquid-filled gap of a characteristic distance. Paired particles exhibit “head-on” alignment, i.e., the particles’ axes of symmetry are aligned with the center-to-center vector (see Fig. 2A), but there is no long-range ordering exhibited between the pairs in the system as shown in the inset of Fig. 2B. The bound pairs then act as a single entity whose motility is drastically reduced as compared to an individual particle because of the orientational persistence of the two particles, as evident in the comparison of their trajectories and mean squared displacements ⟨Δ***r***^2^(Δ*t*)⟩ = ⟨[***r***(*t* + Δ*t*) − ***r***(*t*)]^2^⟩ (Fig. 2C). At longer time scales, the mean square displacement ⟨Δ***r***^2^(Δ*t*)⟩ of both individual particles and particle pairs varies linearly with time. We extract the effective diffusion coefficient *D* for both individual particles and particle pairs through ⟨Δ***r***^2^(Δ*t*)⟩ = 4*Dt* relation and obtain *D*^pair^/*D*^ind^ = 0.32, indicating a substantial reduction in the diffusion of particle pairs compared to the individual particles they are composed of. The nonmotile pairs are capable of inducing further slowing of individual particles around. For the particles slowed by the nonmotile pairs, there is a higher probability of encountering other individual particles and subsequent pair formation. This positive feedback (pair formation and subsequent attraction and trapping of single particles by the pair, resulting in further pair formation) is the main mechanism that drives the emergence of the arrested-motility phase.

**Fig. 3.**
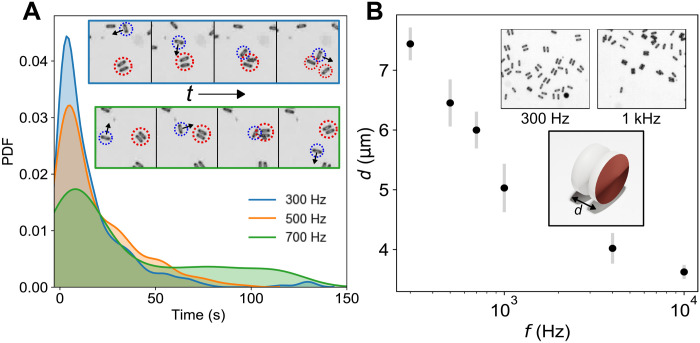
Lifetime of particle pairs and their separation distance frequency dependence. (**A**) Probability distribution function (PDF) of the lifetimes of formed pairs at different input *f*. Inset shows an example of an interaction between an individual particle and a pair at different frequencies *f* = 300 Hz (top) and *f* = 700 Hz (bottom). (**B**) Separation distance (*d*), defined in inset schematic, of the pairs at different pairs. Insets show the microscopy images of several pairs for *f* = 300 Hz and *f* = 1 kHz. The input voltage is 5 V_pp_. Error bars represent SD of the mean.

To investigate in detail the pair dynamics, we use a rules-based algorithm (see Materials and Methods) to automatically detect particle pairs in the recorded sequences of microscopy images (≈5000 frames per single experiment) at different frequencies *f* of the electric field. For frequencies above 300 Hz, we observe an initial growth in the number of pairs formed, followed by a saturation at ≈20 s (Fig. 2D). A formed pair may decay either spontaneously, as one of the particles orients away to leave the pair, or more commonly because of interactions with other particles in the system. Higher frequencies lead to a greater number of pairs forming and sustaining in the system, suggesting increased stability for this configuration at higher *f*. To quantitatively characterize and understand this behavior, we calculate the lifetimes of pairs. The probability density function for the lifetimes of pairs becomes wider for higher *f*, indicating longer living pairs at high frequencies (Fig. 3A). For example, the average lifetime of a pair increases from approximately 15 s at 300 Hz to 38 s at 700 Hz.

To gain insight into the mechanism leading to prolonged life spans at higher *f*, we measure the particle separation distance, *d*, as a function of *f* and reveal that as driving frequency increases, the pair separation distance decreases (see Fig. 3B). The smaller *d* potentially increases the attraction between the paired particles and decreases the decay probability. This phenomenon is illustrated by a series of snapshots in Fig. 3A (inset), where the pair at *f* = 300 Hz (top) is destroyed by a third particle as a result of collision, while a similar pair at *f* = 700 Hz (bottom) remains stable. At lower frequencies, the decay of pairs increases the number of motile individual particles, which, in turn, promote the decay of other pairs. Increased pair stability with the frequency of the driving field allows the system to sustain a higher number of pairs in a steady state as demonstrated in Fig. 2D.

### Theoretical analysis

A nonzero gap between the particles (i.e., the particles are not in steric contact) in a stable pair suggests that interactions mediated by electrical and hydrodynamic fields may play a crucial role. To gain insight into the mechanism of pair formation, we deploy a continuum model for the motion of discoidal ICEP particles that resolves the shape of the particles, the confining solid wall beneath the particles, and the electrical and flow fields in the particles’ vicinity (see Materials and Methods). In particular, the model resolves the frequency dependence of the charging/discharging dynamics of the electrical double layer induced by the AC field.

**Fig. 4.**
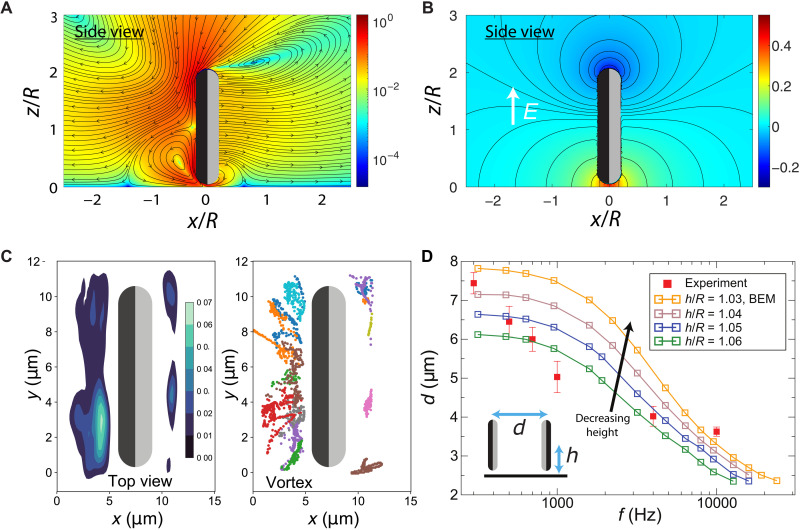
Fields sourced by the particles and pair separation. (**A**) Side view of the flow field in the vicinity of a Janus discoid in the DC limit (*f* → 0). The discoid center is located at *z* = 1.06*R* above the bottom, and the flow field is shown in the symmetry plane *y* = 0. The metal and insulating “faces” of the particle are shown in black and gray, respectively. The color field encodes the magnitude of the fluid velocity, ∣***u***(***x***)∣/*U*_0_. A large vortex is located behind the particle and near the wall. Streamlines start and end on the front and rear of the particle, respectively, reflecting the fact that the particle is moving to the right. (**B**) Disturbance potential ϕ_D_ in the vicinity of the particle from (A). The color encodes the potential in units of *E*_∞_*R*. Black lines show contours of constant potential. The disturbance potential is shown for the background electric field direction indicated by the arrow. (**C**) Experimental data showing the probability distribution (left) and trajectories (right) of tracer particles in the vicinity of a Janus particle. Tracers in the rear vortex move with the particle as they execute recirculating motions in the vortex. (**D**) Steady separation of two particles in a pair as a function of frequency. Closed symbols show the experimental results; open symbols show the results of numerical calculations obtained with the boundary element method (BEM) for different heights above the wall. Error bars are SD of the mean.

As a starting point, we consider the predictions of the model for an isolated particle moving near a planar wall. Figure 4 (A and B) shows the flow **u**(**x**) and disturbance potential ϕ_D_(**x**), respectively, calculated in the DC limit (*f* → 0), where ϕ(**x**) = ϕ_∞_(**x**) + ϕ_D_(**x**) and ϕ_∞_ is the external background potential. Notably, the model predicts formation of a large vortex following the particle that is located near the confining wall. To test this prediction of the model, we introduce tracer particles into the experimental system and track their motion. In agreement with the model, we find a vortex following the active particle (Fig. 4C and movie S2). Moreover, the spatial extent of the observed range of tracer particle attraction shows good agreement with the predicted vortex. The formation of the vortex can be understood as follows. The hydrodynamic flow is being driven around the two “corners” toward the center of the trailing (i.e., metal) face. Necessarily, this creates a stagnation point. To understand the direction of the flow, we consider, without loss of generality, the half-period in which the external field is in the positive *z* direction, as shown by the white arrow in Fig. 4B. Because of polarization of the metal by the external field, the top left corner of the particle acquires a positive surface charge, and the bottom left corner acquires a negative surface charge. Correspondingly, the induced zeta potential is positive on the top left corner and negative on the bottom left corner. For a negative zeta potential, slip is directed from high potential to low potential; for a positive zeta, slip is directed from low potential to high potential (see Eq. 4 in Materials and Methods). Therefore, the slip is directed from the two corners to the center of the metal side of the particle. In addition, the presence of the wall breaks the axisymmetry of the flow and induces recirculation. We note that the flow in Fig. 4A includes a Stokeslet term, representing a force that cancels the nonzero vertical component of the particle’s self-propulsion velocity. However, we find that this term quantitatively, but not qualitatively, affects the flow, as shown in fig. S4, and therefore omit it from the following.

Moving to two-particle interactions, we probe the conditions for formation of bound pairs with finite separation between the particles. We first consider head-on collisions in which both particles are located on the *x* axis and are aligned with the *x* axis. We find that particles can form bound states with finite separation over a broad range of AC-driving frequencies (Fig. 4D). The predicted steady separation *d* decreases for increasing frequency, in qualitative agreement with experiment. Moreover, we find that the presence of the confining planar wall plays a crucial role. To obtain a finite separation over the experimentally relevant range of frequencies, we find *h*/*R* < 1.1, where *R* is the outer radius of the disc and *h* is the height of the particle centroid above the wall (shown schematically in the inset of Fig. 4D). Although the precise height of the particle above the wall is not known, we find broadly similar behavior for different heights *h*/*R* < 1.1. However, the pair separation *d* shifts to smaller values for increasing height.

**Fig. 5.**
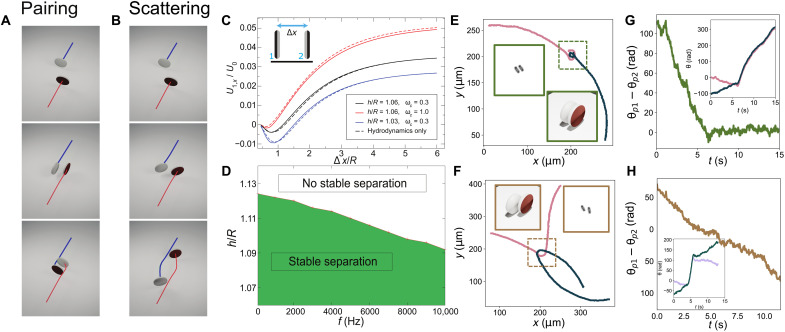
Trajectories and interactions of active particles. (**A**) Trajectories obtained for a small-offset approach between two particles by the theoretical model. (**B**) Trajectories obtained for a high-offset approach between two particles by the theoretical model. (**C**) Velocity of the left particle in a head-on collision (see inset) as a function of center-to-center separation. The steady separation distance *d* is determined by *U*_1,*x*_(*d*) = 0. “Hydrodynamics-only” curves are determined by fixing the surface slip *u*_*s*,*i*_ of a particle at the slip for a single particle near a wall. (**D**) Phase map showing the conditions for a stable and finite separation in a head-on collision. In the white region, particles approach each other until contact. (**E**) Representative pairing trajectory observed in experiments for two particles with a small-offset approach. Insets are a snapshot and three-dimensional (3D) representation of the two particles at the moment of approach. (**F**) Representative nonpairing trajectory observed in experiments for two particles with a high-offset approach. Insets are a snapshot and 3D representation of the two particles at the moment of approach. (**G**) Difference between the orientation angles of the two particles for a small-offset approach. Inset is the orientation angles plotted separately for the two particles. (**H**) Difference between the orientation angles of the two particles for a high-offset approach. Inset is the orientation angles plotted separately for the two particles *E* = 0.13 V μm^−1^ and *f* = 500 Hz.

To test whether the model reproduces the experimentally observed alignment of two particles, we consider “off-center” collisions in which two particles are initially aligned with the *y* axis but separated in both the *x* and *y* directions. We fix the particle height as *h*/*R* = 1.06. For an initial offset in the *y* direction Δ*y*/*R* = 5, we find that for an initial offset in the *x* direction, 0 ≤ Δ*x*/*R* ≲ 1.5, the two particles will form a head-on bound state (Fig. 5A). For an initial Δ*x*/*R* ≳ 1.5, we obtain scattering trajectories where the particles pivot around each other and leave without forming a pair (Fig. 5B).

Next, to determine the dominant physical mechanism producing the finite separation, we consider the velocity of one of the colliding particles as a function of separation (Fig. 5C). The solid curves show the predictions of the model for various heights *h*/*R*. We also consider a “hydrodynamics-only” modification of the model in which the distribution of ICEP slip at the surface of a particle (see Materials and Methods) is fixed at the distribution for an isolated particle (dashed curves). This approach treats particles as an effective “squirmer” and neglects variation of the surface slip induced by the effect of the second particle on the electric field. We find that the predictions of the hydrodynamics-only model show close agreement with those of the full model. This finding indicates that hydrodynamic interactions dominate the mechanism of pair formation. In addition, for each of the three conditions shown in Fig. 5C, there is a stable head-to-head separation distance greater than the distance of steric contact, i.e., with a liquid-filled gap between the particles. In Fig. 5D, we show that, over the range of frequencies considered here, there is always a finite and stable separation distance when the particles are sufficiently close to the wall. Thus, confinement plays an essential role in pair formation.

Experimentally, we can compare the collision conditions for two particles when they approach each other and in agreement with the predictions of the model for a small off-center distance, we find that they create a pair with a finite separation distance (Fig. 5E and movie S3). In the case where particles approach each other with a high off-center distance (>*r*), particles do not form a stable pair but pivot and leave each other (Fig. 5F and movie S4). Plotting θ_*p*1_ − θ_*p*2_, we see convergence close to 0 at *t* = 6 s for the small-offset collision case (Fig. 5G) but do not observe any convergence when the particles approach each other at a high off-center distance (Fig. 5H). The particles also do not form a stable pair if the collision angles are highly misaligned from a head-on collision. We find that the difference in approach angles needs to be close to a head-on collision (2π/3 < θ_*p*1_ − θ_*p*2_ < 4π/3) for pair formation to occur. This orientational alignment behavior observed in shape-anisotropic discoidal particles is radically different from when two spherical ICEP particles approach each other head on. In this case, they pause at a finite separation distance similar to discoidal particles, but this state is unstable against rotation of particle orientations causing the particles to slide past each other (section S2). Spherical particles may form a stable pair if their sizes are substantially different (*28*, *29*). Consequently, even at higher particle densities, systems of spherical particles do not have an arrested-motility phase, indicating that shape anisotropy plays a crucial role in the formation of this phase in discoidal particles.

Overall, the findings of the numerical model indicate that hydrodynamic interactions play a dominant role in producing head-on alignment and finite pair separation. However, the detailed hydrodynamic mechanism of alignment, and particularly the role of particle shape, remains unclear. From previous work, dating back to the classic work of Jeffery (*30*) on particle motion in simple shear flow, it is known that prolate spheroids, oblate spheroids, and spheres show distinct rotational behavior in the presence of velocity gradients through a distinction in how they couple to the rate-of-strain component of the flow. Accordingly, we hypothesize that alignment for the discoids is driven by such a coupling. To show that this mechanism can produce the alignment, we develop a minimalistic, “point-particle” model of two hydrodynamically interacting swimmers. The details of the model and calculations are provided in Materials and Methods. Here, we highlight two key points: First, the orientation ${\hat{d}}_{i}$ of particle *i* evolves according to

$${\overset{\cdot }{\hat{d}}}_{i}=(I-{\hat{d}}_{i}{\hat{d}}_{i})\cdot (\Gamma E(x_{i})+W(x_{i}))\cdot {\hat{d}}_{i}$$ (1)

where the shape parameter Γ is negative for an oblate particle, zero for a sphere, and positive for a prolate particle; I is the identity tensor; **E** is the rate-of-strain tensor; and **W** is the vorticity tensor. Second, the point-particles interact via a hydrodynamic force-dipole characterized by the parameter σ_0_, which is negative for “pushers” and positive for “pullers.” As detailed in Materials and Methods, we show that the point-particles have head-on bound state that is stable against perturbation of the particles’ orientations if Γ < 0 (e.g., for discoidal shapes) and σ_0_ < 0 (i.e., the particles are pushers). This finding explains the key role of the discoidal shape of the particle. Note that from Fig. 4A, it can be inferred that the ICEP particles are pushers, as the surface slip is localized to the rear (i.e., metal side) of the particles, where the induced zeta potential is nonzero. As additional illustration, we show an off-center collision that leads to a bound state in fig. S5 obtained with the point-particle model.

**Fig. 6.**
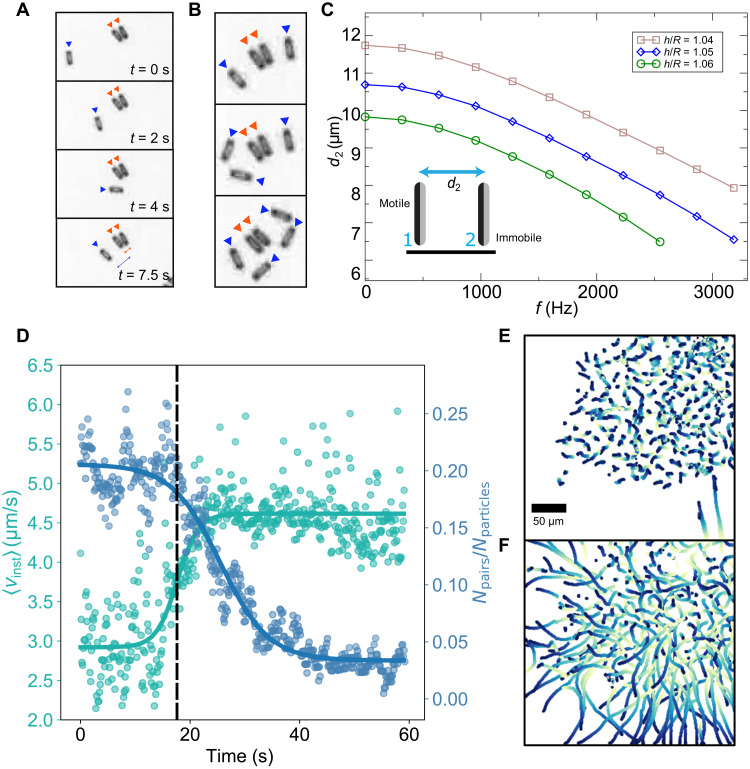
Reversibility of arrested-motility phase in systems of discoidal Janus active particles. (**A**) Snapshots showing the approach and alignment of an individual motile particle (blue marker) on the metal side of a particle pair (red markers). The arrows in the bottom show the separation distance between paired particles (≈5 μm) and individual particle and the paired particle (≈11 μm). (**B**) Snapshots of the different configurations resulting from the interactions between individual particles and pairs. (**C**) Steady separation *d* of bound pairs that have an insulator-to-metal configuration, obtained in numerical calculations for different heights. Notably, for fixed height and frequency, the separation is greater than for the head-on configuration (compare with Fig. 4D). (**D**) Time evolution of average ensemble velocity (left axis) and number of pairs (right axis) in the system over the same time period. The dotted line at *t* = 18 s represents the point at which frequency is switched from *f* = 500 Hz to *f* = 250 kHz. (**E** and **F**) Tracked trajectories of particles before and after switching to the sDEP mechanism. Color code represents progression of time.

The arrested-motility phase develops in the system directly as a result of the pair formation phenomenon. In addition to the reduced motility of a pair due to the orientational persistence of the two particles, the pairs attract additional particles in their vicinity through both collision interactions and, more importantly, an active alignment interaction. When an individual active particle approaches the metal (outer) side of one of the particles in the pair (Fig. 6A), it tends to align with the bound pair at a finite separation distance. This phenomenon can also occur for individual stuck particles, i.e., motile particles that approach the immobile particle on the metal side become orientationally coupled, in addition to the strong pairing mechanism that can occur on the insulator side. This two-body interaction is more amenable to theoretical analysis. This pair-particle separation distance is larger than the separation distance between the particles in a paired configuration (Fig. 6A, bottom). Likewise, in simulations of a mobile particle approaching a stalled particle from the rear, we obtain a stable separation that is larger, for a given AC frequency and particle height, than for head-on collisions (Fig. 6C and movie S5). In the experiments, we also observe configurations where more than one particle can interact and become nonmotile on either side of the pair (Fig. 6B). This orientational coupling on the metal side is weaker than on the insulator side (i.e., paired particles), which leads to individual particles occasionally leaving the configurations shown in Fig. 6B. Overall, areas with a large number of particle pairs also have a higher number of individual particles around them, which can lead to further pair formation in the area due to increased probability of particle-particle encounters. This reinforcing mechanism of pair formation and increased concentration of individual particles around the formed pairs leads to increased particle density and arrested motility in certain areas of the experimental cell as confirmed by the tracked particle trajectories (Fig. 6E). Orientational coupling between individual motile particles and pairs occurs only when the individual particles are in close proximity of the pair, and there is no long range attractive force, which makes motility a key ingredient for the development of this phase. The increased density of particles through pair formation in these discoidal particles differs from the compact clusters observed for the case of self-diffusiophoretic active particles where diffusiophoretic attraction between particles leads to clustering (*21*, *31*). Groups formed via particle pair formation are sparse, and the particles participating in the group do not actually touch each other.

### Reversibility of the arrested-motility phase

The arrested-motility phase formed via pair formation is also reversible. When the frequency of the applied field exceeds the charge relaxation time of the electric double layer around the particle, the induced charge flows and ICEP mobility decay to zero. At higher frequencies (100-kHz to 10-MHz range), self-propulsion is enabled by a completely different phenomenon and in the opposite direction, toward their metallic side (*32*). A nonuniform electric field induced by the dual asymmetry of the particle and the confining wall acts on the dipole within the Janus particle to drive net motion, a mechanism termed as self-dielectrophoresis (sDEP) (*32*). In the sDEP regime, a repulsive interaction between particles comes into effect, and the particles tend to avoid each other and no longer form pairs.

When the driving frequency is changed to *f* = 250 kHz in our experiment, the system goes from being dominated by ICEP mechanism (arrested-motility phase) to that of sDEP mechanism where the formed pairs are destroyed. Consequently, the motility in the system increases with the average ensemble velocity 〈*v*_inst_〉 changing from 〈*v*_inst_〉 ≈ 3 μm/s to 〈*v*_inst_〉 ≈ 4.5 μm/s, while simultaneously, the number of pairs in the system decreases (Fig. 6D and movie S6). The trajectories are no longer confined as the particles propel away from the group, and the system returns to a uniform distribution of particles (Fig. 6, E and F).

## DISCUSSION

In conclusion, we demonstrated a new active matter system consisting of self-propelled disk-shaped microparticles energized by an AC electric field and driven by ICEP mechanism. Shape asymmetry of the synthesized particles introduces controlled complexity to their individual and collective dynamics. At high particle densities, an arrested-motility phase develops where the particle trajectories are highly confined and the system motility is drastically reduced. This collective effect results from a positive feedback mechanism of particles slowing down through hydrodynamics-induced pair formation and the further formation of pairs through a secondary slow-down effect of particles near particle pairs.

We show that shape asymmetry plays an important role in this response. It promotes formation of particle pairs, seeding further local accumulation of particles and ultimately leading to a loose cluster phase with arrested motility. The arrested-motility phase is dynamic and reversible through the parameters of the driving field. A detailed numerical model of interacting ICEP particles shows good agreement with the observed pairing dynamics, including reduction of the pair separation distance with increasing frequency of the driving electric field.

Intriguingly, our findings bear similarities to recent work on so-called torque-induced aggregation, in that head-to-head or head-to-tail alignment of particle pairs promotes formation of loose and fluid clusters (*33*). However, detailed analysis of our system reveals important distinctions. While the particles in *33* are spherical and interact through electrostatic torques, in our system, interactions between particles are dominated by hydrodynamics. Effectively, the particles act as discoidal squirmer particles with field-tunable propulsion speed and force dipole. In turn, this finding motivated the development of a simple point-particle model of interacting squirmers with oblate shape. This model has successfully captured the observed pairing dynamics, revealing that the key ingredient in the pair formation dynamics is how the oblate shape of a particle couples to ambient fluid velocity gradients generated by other particles. The effect of nonspherical shape on the dynamics of squirmers is an emerging area of study (*34*–*37*). To our knowledge, it was not previously shown that oblate or discoidal squirmers can form bound pairs. Our prediction of a robust pair formation in systems of oblate/discoidal squirmers is generic and should carry over to other experimental systems that can be described with the squirmer model (*38*).

Although our modeling and theoretical analysis focuses on pair formation, future works could explore the role of many-body interactions in the dynamics of the system. Our simulation method could be applied to the interaction of several particles without much additional computational expense. However, for large systems of particles, a coarse-grained technique would be needed. Various methods available in the literature are potentially suitable for this problem (*39*–*41*). In addition, the point-particle model developed here could be, in principle, adapted to large systems. A more sophisticated version of this approach, accounting for the finite size of the particles, would be equivalent to active Stokesian dynamics (*42*).

## MATERIALS AND METHODS

### Experimental details

The discoidal particles (10 μm in diameter) are fabricated of negative photoresist (SU-8) via direct laser writing technique. A thin 25-nm metal layer of gold is thermally sputtered on one side of the disks. The particles are suspended in a 0.1% Tween 20 surfactant solution, which minimizes particle aggregation and prevents particles from sticking to surfaces. A droplet of the particle suspension is sandwiched between two conductive indium tin oxide glass slides separated by a spacer (*h* = 60 μm) and a voltage is applied using a function generator.

### Rules-based algorithm for detection of paired particles

Particle locations and trajectories are extracted using an automated program developed in-house. The Python-based program uses Open CV for image processing and uses a threshold segmentation method to obtain particle centroid locations and standard Bayesian decision-making algorithms to calculate particle trajectories. For every particle in the system, for every frame, we calculate the Euclidean distance *d*(*p*, *p_n_*) and relative angle (∣θ − θ*_n_*∣) to all other particles. We then apply alimiting rule of *d*(*p*, *p_n_*) < 10 μm and ∣θ − θ*_n_*∣ < 30° to determine whether two particles belong to a pair. We label two particles as belonging to a pair if the above conditions are fulfilled for at least *t* = 60 s.

### Theoretical model

#### Continuum model

We rationalize the pairing and scattering dynamics of two particles by modeling particle motion driven by the surface-induced coupling of the AC electric field and hydrodynamic flow (*43*–*47*). Specifically, we adapt the model presented by Kilic and Bazant (*45*) for the dynamics of an ICEP sphere near an insulating planar wall. Here, we consider two discoidal metallo-dielectric ICEP particles immersed in liquid solution above a conducting planar wall. The wall is located at the position *z* = 0 and has a normal in the $\hat{z}$ direction. The geometric centroid of a particle *i* ∈ {1,2} is located at position **x***_i_*. Each particle has a dielectric “face” and a metal face. The outer radius of a particle is *R*. The orientation of particle *i* is defined by the vector ${\hat{d}}_{i}$, which points along the axis of the particle from the metal face to the inert face. The particles are immersed in a Newtonian solution with electrical permittivity ϵ and dynamic viscosity μ.

The particles are exposed to a externally imposed, uniform AC electric field in the $\hat{z}$ direction with amplitude *E*_∞_ and angular frequency ω. We take ϕ_0_ ≡ *E*_∞_*R* to define a characteristic scale for the potential and write the dimensionless electric potential in the vicinity of the particles as ϕ(**x**, *t*). The characteristic time scale for particle motion is defined as $t_{0}\equiv \mu /\epsilon E_{\infty }^{2}$. If *t*_0_ is much longer than the period of the external AC field, then one can approximate the particle as being stationary over one period. Accordingly, following Kilic and Bazant, we consider the complex-valued, period-averaged dimensionless potential $\tilde{\phi }(x)$, as defined by $\phi \equiv \text{Re}(\tilde{\phi }e^{i\omega t})$. The field $\tilde{\phi }$ obeys the Laplace equation $\nabla^{2}\tilde{\phi }=0$ in the fluid domain, the boundary condition $\hat{n}\cdot \nabla \tilde{\phi }=i\omega_{c}\tilde{\phi }$ on the conducting face of a particle, $\hat{n}\cdot \nabla \tilde{\phi }=0$ on the insulating face, $\hat{n}\cdot \nabla \tilde{\phi }=-1$ on the planar wall, and $\nabla \tilde{\phi }\to -\hat{z}$ as ∣**x**∣→∞. The parameter ω*_c_* is a dimensionless frequency defined as ω*_c_* ≡ ωτ*_c_*, where the characteristic charging time for a double layer τ*_c_* = λ_D_R/*D*, *D* is the diffusivity of ions, and λ_D_ is the Debye length. As discussed in detail in (*45*), the first-order boundary condition on the conducting surface of a particle represents the capacitive charging and discharging of the double layer surrounding the particle surface in response to the induced surface charge. In principle, a Robin boundary condition should also hold on the metal electrode. However, the Green’s function for this boundary condition requires laborious numerical integration (*48*), and the Neumann boundary condition should be approximately valid for low frequencies.

The hydrodynamic flow is governed by the Stokes equation

$$-\nabla p+\mu \nabla^{2}u=0$$ (2)

where **u**(**x**) is the velocity field and *p*(**x**) is the pressure field. The solution is assumed to be incompressible, i.e., ∇ · **u** = 0, and to have uniform density and viscosity. The velocity field obeys the boundary condition

$$u=U_{i}+\Omega_{i}\times (x-x_{i})+u_{s,i}(x_{s})$$ (3)

on the surface of the particle *i* ∈ {1,2}, and **u** = 0 on the planar wall, assumed to be no slip. Here, **U***_i_* is the (unknown) translational velocity of particle *i*, **Ω***_i_* is the (unknown) angular velocity of particle *i*, and **u**_*s*,*i*_(**x***_s_*) is the so-called hydrodynamic slip at a point **x***_s_* on the surface of a particle. This quantity is given by

$$u_{s,i}=\frac{1}{2}\text{Re}({\tilde{\zeta }}_{i}\nabla_{\|}{\tilde{\phi }}^{*})$$ (4)

where the surface gradient operator is $\nabla_{\|}=(I-\hat{n}\hat{n})\cdot \nabla$, where I is the identity tensor, and

$${\tilde{\zeta }}_{i}(x_{s})=g(x_{s})[C_{i}-\tilde{\phi }(x_{s})]$$ (5)

is the induced zeta potential. The zeta potential is the difference in electric potential between the “shear plane” and the bulk solution. The shear plane is defined as the boundary between the bulk solution and liquid (including dissolved ions) bound to the particle surface. The function *g*(**x***_s_*) is defined as *g*(**x***_s_*) = 1 on the metal face of a particle and *g*(**x***_s_*) = 0 on the dielectric face. The constant *C_i_* ensures that there is no net induced charge over the surface of the particle *i*. Because we consider period-averaged particle motion induced by AC electric fields, we can neglect the equilibrium zeta potential. It is determined as

$$C_{i}=\int_{S_{i}}g(x_{s})\;\tilde{\phi }(x_{s})\;\mathrm{dS}/\int_{S_{i}}g(x_{s})\;\mathrm{dS}$$ (6)

where the integrals are taken over the surface *S_i_* of particle *i*. The particles are assumed to be force-free and torque-free, i.e.

$$\int_{S_{i}}\sigma \cdot \hat{n}\;\mathrm{dS}=0$$ (7)

and

$$\int_{S_{i}}(x-x_{i})\times (\sigma \cdot \hat{n})\;\mathrm{dS}=0$$ (8)

where **σ** = μ(∇**u** + ∇**u***^T^*) is the stress tensor for a Newtonian fluid. The force-free and torque-free conditions close the system of equations for the unknowns **U***_i_* and **Ω***_i_*.

We solve the Laplace equation numerically, using the boundary element method (*49*). Specifically, we solve for the (dimensionless) disturbance potential ${\tilde{\phi }}_{D}$, where $\tilde{\phi }\equiv {\tilde{\phi }}_{\infty }+{\tilde{\phi }}_{D}$, and ${\tilde{\phi }}_{\infty }=-z/R$. Rather than solve the Stokes equation directly, we use the Lorentz reciprocal theorem. Specifically, we consider 12 auxiliary problems. Each of the auxiliary problems *j* corresponds to a mode of motion of the system, i.e., translation of one of the particles with unit velocity in the three Cartesian directions or rotation of one of the particles with unit angular velocity around an axis in one of the three Cartesian directions. The solution to auxiliary problem *j* yields a velocity field **u**^(*j*)^ and a stress field **σ**^(*j*)^. We define a generalized velocity **V** as **V** ≡ (**U**_1_, **U**_2_, **Ω**_1_, **Ω**_2_)*^T^*. It can be shown that **V** satisfies 6πμRV=b, where R is the two-particle grand resistance tensor and the component *j* of ***b*** is

$$b_{j}=\int_{S_{1}}u_{s,1}\cdot \sigma^{\left(j\right)}\cdot \hat{n}\;\mathrm{dS}+\int_{S_{2}}u_{s,2}\cdot \sigma^{\left(j\right)}\cdot \hat{n}\;\mathrm{dS}$$ (9)

To compare our model with experiment, we must estimate τ*_c_*. Taking R = 5 μm, λ_D_ = 10 nm, and *D* ∼ 10^−9^ m^2^/s for most ions in solution, we obtain τ*_c_* = 5 × 10^−5^ s. The particle and flow velocities are nondimensionalized by the characteristic velocity $U_{0}\equiv \epsilon E_{\infty }^{2}R/\mu$. We have validated our numerical model by recovering the results in (*45*) for the force on a spherical conducting particle near an insulating wall. The model also allows calculation of the Maxwell stress on particles, but we found it to be negligible for the frequency range considered.

We simplify the dynamics with the following assumptions. We assume that the particles have fixed height *h* above the planar wall and that their orientations ${\hat{d}}_{i}$ are oriented in the plane of the wall. We perform two kinds of calculation. First, we consider “head-on collisions” of two particles that have ${\hat{d}}_{1}=-{\hat{d}}_{2}$ and ${\hat{d}}_{1}$ parallel to the center-to-center separation vector **x**_12_ ≡ **x**_2_ − **x**_1_. In other words, their dielectric faces point toward each other. In these calculations, we consider whether the particle pair achieves a stable and finite separation, i.e., a bound state, as observed in the experiments.

In the second kind of calculation, we consider off-center collisions in which the particles initially have ${\hat{d}}_{1}=-{\hat{d}}_{2}=-\hat{y}$, but the initial *x* position of particle 2 is offset from the initial *x* position of particle 1. From these initial conditions, we perform “on-the-fly” numerical integration of the particles’ rigid body dynamics, using quaternions to track the particles’ orientations (*34*). In these calculations, the particles are free to move in the *xy* plane and rotate around the $\hat{z}$ axis. Accordingly, these rigid body dynamics calculations probe the stability of a bound state with respect to rotation of the particles and lateral translation of the particles. In addition, they allow us to simulate fully two-dimensional pair formation and scattering dynamics.

#### Point-particle model

The finding that hydrodynamics dominates particle interactions suggests that the observations can potentially be recovered within a simpler model. Here, we consider the interaction of two microswimmers using a minimal point-particle model. Specifically, the particles move with velocity

$$U_{1}=U_{s}\;{\hat{d}}_{1}+u(x_{1})$$ (10)

$$U_{2}=U_{s}\;{\hat{d}}_{2}+u(x_{2})$$ (11)

where U_s_ is the speed of an isolated swimmer and **u**(**x**_α_) represents the perturbation to the ambient flow at the position of swimmer α due to the other swimmer. Each swimmer α is associated with a hydrodynamic stresslet

$$S_{\alpha }=\sigma_{0}({\hat{d}}_{\alpha }{\hat{d}}_{\alpha }-\frac{I}{3})$$ (12)

Here, σ_0_ is the strength of the force-dipole governing far-field hydrodynamic interactions. For a pusher, σ_0_ < 0, while for a puller, σ_0_ > 0. Note that the stresslet is traceless by construction. The orientations of the two swimmers change according to (*50*)

$${\overset{\cdot }{\hat{d}}}_{1}=(I-{\hat{d}}_{1}{\hat{d}}_{1})\cdot (\Gamma E(x_{1})+W(x_{1}))\cdot {\hat{d}}_{1}$$ (13)

$${\overset{\cdot }{\hat{d}}}_{2}=(I-{\hat{d}}_{2}{\hat{d}}_{2})\cdot (\Gamma E(x_{2})+W(x_{2}))\cdot {\hat{d}}_{2}$$ (14)

Here, Γ is a shape parameter that is zero for a sphere, positive for a prolate spheroid (which has its major axis aligned with $\hat{d}$), and negative for an oblate spheroid. The second-rank tensors **E**(x_α_) and **W**(x_α_) represent the rate of strain and vorticity, respectively, evaluated at x_α_, where

$$E=\frac{1}{2}(\nabla u+\nabla u^{T})$$ (15)

and

$$W=\frac{1}{2}(\nabla u-\nabla u^{T})$$ (16)

In index notation, the velocity field created by a stresslet **S** is

$$u_{i}=\frac{1}{8\pi \mu }(\frac{x_{i}\delta_{\text{jk}}}{r^{3}}-\frac{3x_{i}x_{j}x_{k}}{r^{5}})S_{\text{jk}}$$ (17)

where **x** is the displacement vector between the position of the stresslet and a location in the surrounding fluid, and *r* =∣**x**∣(*51*). It follows that, using that *tr*(**S**) = 0

$$\frac{\partial u_{i}}{\partial x_{\ell }}=\frac{1}{8\pi \mu }(-\frac{3x_{j}x_{k}\delta_{i\ell }S_{\text{jk}}}{r^{5}}-\frac{3x_{i}x_{k}S_{\ell k}}{r^{5}}-\frac{3x_{i}x_{j}S_{j\ell }}{r^{5}}+\frac{15x_{i}x_{j}x_{k}x_{\ell }S_{\text{jk}}}{r^{7}})$$ (18)

We now consider the head-on collision of two swimmers. Swimmer 1 is located at (−Δ,0,0) and has orientation ${\hat{d}}_{1}=\hat{x}$. Swimmer 2 is located at (Δ,0,0) and has orientation ${\hat{d}}_{2}=-\hat{x}$. It is easily shown that the pusher swimmers have a steady separation

$$d=2\Delta^{*}=\sqrt{\frac{-\sigma_{0}}{4\pi \mu U_{s}}}$$ (19)

For this head-on configuration, **W**(**x**_1_) = 0 and **W**(**x**_2_) = 0. Thus, for a small perturbation in the orientation of swimmer α, we expect the angular velocity of swimmer α to be determined by the quantity $\Gamma E(x_{\alpha }^{*})$, where $x_{1}^{*}=(-\Delta^{*},0,0)$ and $x_{2}^{*}=(\Delta^{*},0,0)$. Focusing on swimmer 1, we write

$${\hat{d}}_{1}=(\text{cos}\;(\phi ),\text{sin}\;(\phi ),0)$$ (20)

and obtain

$$-\text{sin}\;\phi \;\overset{\cdot }{\phi }=\Gamma [E_{\text{xx}}\text{cos}\;\phi \;{\text{sin}}^{2}\phi +E_{\text{xy}}\;\text{sin}\;\phi (1-2{\text{cos}}^{2}\phi )-\text{cos}\;\phi \;{\text{sin}}^{2}\phi \;E_{\text{yy}}]$$ (21)

For the head-on configuration under consideration, we obtain *E_xy_* = 0, $E_{\text{xx}}=\frac{3S_{\text{xx}}}{32\pi \mu \Delta^{2}}$, and *E_yy_* = − *E_xx_*/2. Inserting *S_xx_* = 2σ_0_/3, we obtain, for ϕ ≠ 0

$$\overset{\cdot }{\phi }=-\frac{3\Gamma \sigma_{0}}{32\pi \mu \Delta^{2}}\text{cos}\;\phi \;\text{sin}\;\phi$$ (22)

As expected, the expression has nematic symmetry in ϕ. Thus, the stability of the bound state depends on the sign of Γσ_0_. Because we deduced σ_0_ < 0 (i.e., the particles are pushers) to obtain a real-valued stable separation, it follows that Γ < 0, i.e., the particle has an oblate or discoidal shape.

## Supplementary Materials

### This PDF file includes:

Sections S1 to S5 Figs. S1 to S5
