# Supplementary material for: Arrested-motility states in populations of shape-anisotropic active Janus particles
Source: Sci Adv. 2022 Jul 1;8(26):eabo3604. doi: 10.1126/sciadv.abo3604 (PMC10883369; doi:10.1126/sciadv.abo3604)
Supplement: Supplementary file 1 — Sections S1 to S5 Figs. S1 to S5 [file sciadv.abo3604_sm.pdf]

Science Advances
AAAS

Supplementary Materials for

Arrested-motility states in populations of shape-anisotropic active Janus particles

Jaideep Katuri *et al.*

Corresponding author: Alexey Snezhko, snezhko@anl.gov

Sci. Adv. **8**, eabo3604 (2022)
DOI: 10.1126/sciadv.abo3604

The PDF file includes:

Sections S1 to S5
Figs. S1 to S5
Legends for movies S1 to S6

Other Supplementary Material for this manuscript includes the following:

Movies S1 to S6

I. INDIVIDUAL BEHAVIOR OF SHAPE ANISOTROPIC ACTIVE PARTICLES

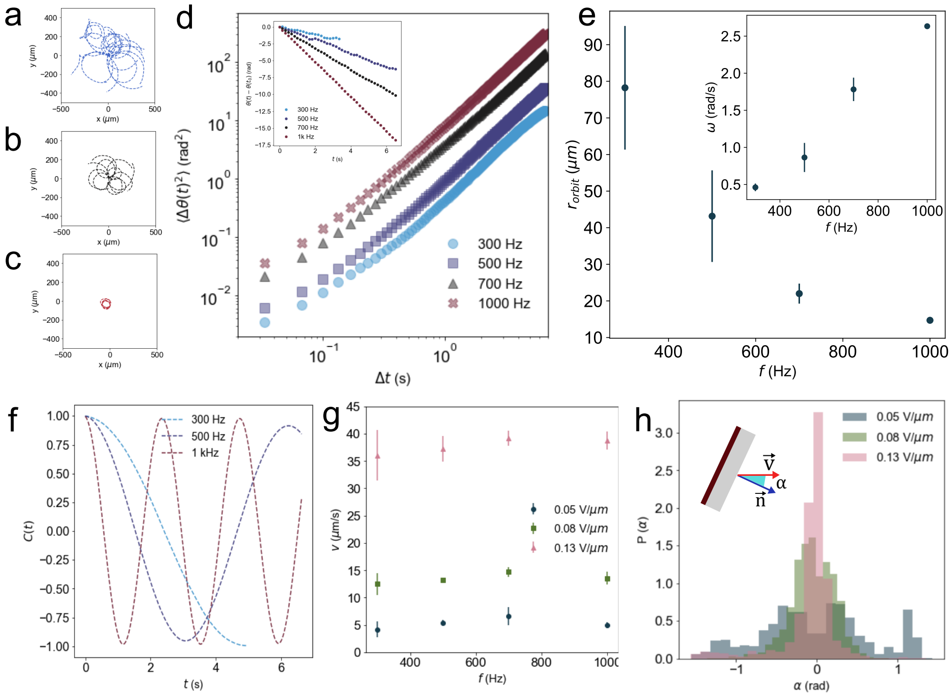

FIG. S1. **Individual behavior of shape anisotropic active Janus particles.** Example trajectories of active particles over 10 s for the driving frequency (f) of (a) 300 Hz, (b) 500 Hz and (c) 1000 Hz and electric field amplitude of (E) = 0.13 $V\mu m^{-1}$. (d) Mean squared angular displacement (MSAD) calculated from particle orientations (θ) ($E = 0.13\ V\mu m^{-1}$). Inset in (d) shows the time evolution of the in-plane angle θ ($E = 0.13\ V\mu m^{-1}$). (e) Average orbital radius of particle trajectories for different f ($E = 0.13\ V\mu m^{-1}$). Inset is the average angular velocity (ω) of particles at different frequencies. (f) Angular autocorrelation $C(t)$ of the active particles for different frequencies.($E = 0.13\ V\mu m^{-1}$) (g) Self-propulsion velocity (v_p) of the Janus disks for different f and E. (h) Probability distribution of offset angle (α) for three amplitudes of the electric field E

We analyze the individual behavior of the disk-shaped active particles at low frequencies (< 10 kHz) where the particles move towards their dielectric side by the ICEP mechanism described above. Unlike spherical particles, the disk shaped particles move along circular trajectories. The curvature of the trajectories increases with the frequency of the applied field f, Fig. S1(a-c). The direction of the rotation is chosen randomly, therefore clockwise and anti-clockwise trajectories are observed at equal probabilities. While particles do not reverse the direction of rotation spontaneously, the reversal may be triggered by interactions with other particles in the system.

The circular motion of particles can be tracked and characterized by its orientation angle θ. The orientation of a particle $\mathbf{n}$ is defined as a normal to the dielectric side of the disk. We quantify the rotational behavior of the particles by measuring their mean squared angular displacement (MSAD) $\langle \Delta\theta(t)^2 \rangle = \langle [\theta(t+t_0) - \theta(t_0)]^2 \rangle$ at different frequencies f of AC field but at a fixed amplitude (Fig. S1(d)) which allows us to introduce the angular velocity ω of particle motion. According to our measurements, ω increases with driving field frequency f, for example, from ω = 0.46 rad/s at f = 300 Hz to ω = 2.63 rad/s at f = 1 kHz for $E = 0.13\ V\mu m^{-1}$ (Fig. S1(e) inset) and the orbital radius reduces from $r_{orbit} = 78.23\ \mu m$ to $r_{orbit} = 14.71\ \mu m$ for the same frequency range (Fig. S1(e)).

Another quantitative characterization of particle motion can be done through the angular autocorrelation function defined as $C(t) = \left\langle \frac{\mathbf{v}(t_0+t)\cdot\mathbf{v}(t_0)}{|\mathbf{v}(t_0+t)||\mathbf{v}(t_0)|} \right\rangle_{t_0}$. For example, the angular autocorrelation is constant for a purely ballistic swimmer and exponentially decaying function for a diffusive swimmer. In our system of active disk swimming in a circular trajectories $C(t)$ oscillates with a characteristic time T' that is inversely proportional to the driving frequency f, see Fig. S1(f).

The ability to control the rotational behavior of the active particles directly (through the driving field frequency) is an important feature of our system which eliminates the need for an additional field to apply the rotational torque. Moreover, the linear and rotational motions are decoupled. While the driving field frequency f controls the rotation of a particle, the amplitude of the field E prescribes the linear speed or particle's velocity, as shown in Fig. S1(g). With the increase of E the difference in polarization between the two sides of the disk-shaped particle is enhanced resulting in faster v_p, similar to the behavior of spherical particles. In contrast with spherical particles, the disk-shaped particles are more directional at higher E, which is manifested by a smaller offset angle α between the orientation of the particle $\mathbf{n}$ and the direction of the velocity vector ($\mathbf{v}$), see Fig. S1(h) inset. The probability distribution of α is broad in the case of low E, but has a sharp peak at ≈ 0 for higher E.

II. EXPERIMENTAL RESULTS FOR SPHERICAL JANUS PARTICLES

Spherical ICEP janus particles are prepared by deposition a layer of Ti (25 nm) on spherical silica particles (10 μm). The particles are suspended in a similar set-up to the one used for disk shaped particles (sandwiched between conductive ITO glasses) and an AC electric field is applied using a function generator. Before activation we find that the particles are suspended with their caps-down, appearing as dark circles under an optical microscope, due to the bottom heaviness of the metal cap. Once an AC field is applied the particles switch their orientation such that their symmetry axis is parallel to the bottom substrate. At low frequencies (¡ 10 kHz), the particles self-propel away from the metal cap. We record interactions between two spherical particles approaching head-on at 500 Hz and find that spherical particles pause at a finite distance but this configuration is unstable against the rotation of particle orientations and the particles slide past each other (Fig. S2).

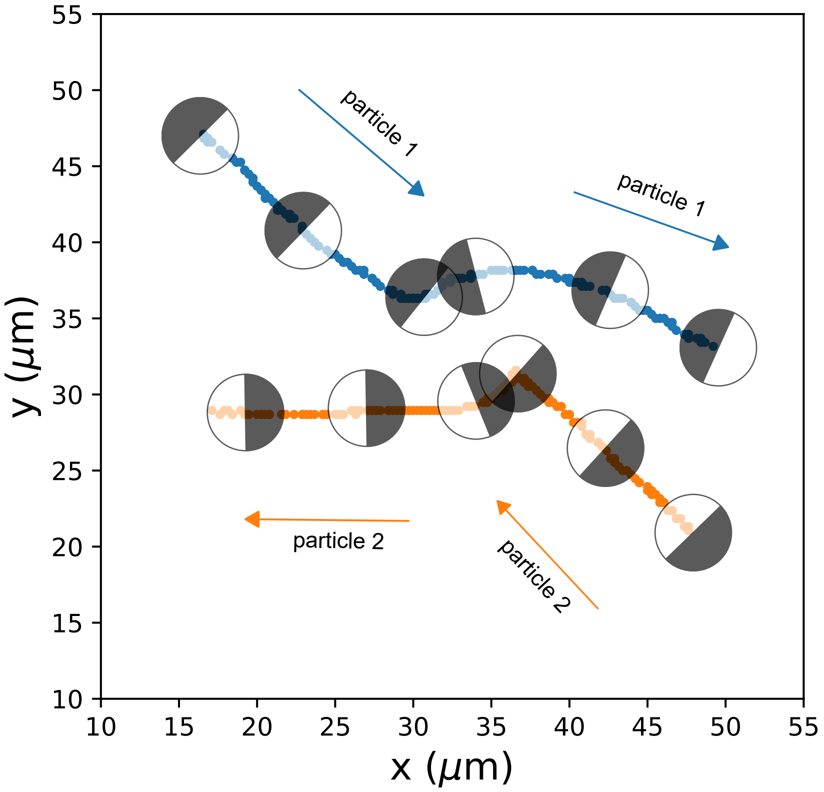

FIG. S2. **Representative particle trajectories for two spherical ICEP particles approaching head-on.**

III. SIMULATION RESULTS FOR SPHERICAL JANUS PARTICLES

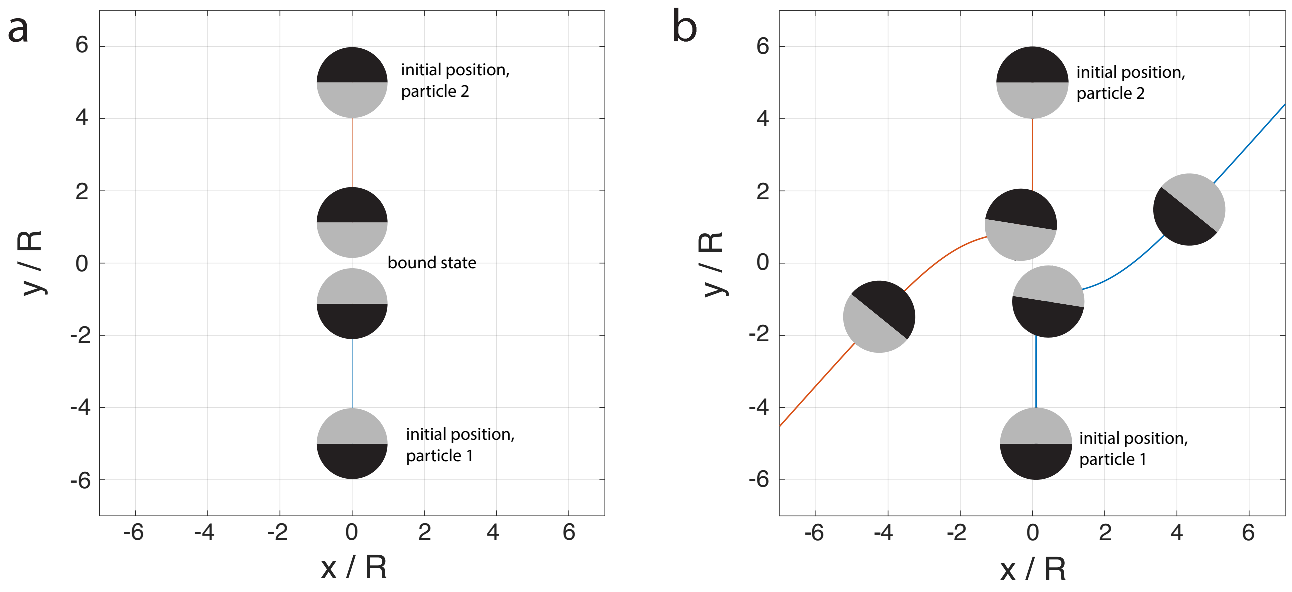

FIG. S3. **Simulated particle trajectories for two spherical ICEP particles.** (a) Head-on collision of two spherical particles, obtained with the BEM in the DC limit. The particles form a bound state with a non-contact separation. The particle heights are fixed as $h/R = 1.06$. (b) The same system as in (a), but with an slight initial lateral separation of $\Delta x/R = 0.1$. The particles slide past each other and scatter.

Applying the numerical model to spherical particles, we find that there is a head-on bound state with finite separation, but it is unstable against angular or lateral perturbations. In Fig. S3a, we show that two spheres in a head-on bound state achieve a finite separation. However, in Fig. S3b, we apply a slight lateral perturbation ($\Delta x/R = 0.1$) to the initial configuration of one of the particles. Consequently, the particles "slide" past each other.

IV. COMPARISON OF FLOW FIELDS WITH AND WITHOUT STOKESLET TERM

The microscopic model can yield non-zero values of the vertical component of the particle velocity U_z. When the particle is oriented vertically with respect to the wall (i.e., $\hat{d}$ is in the plane of the wall), U_z is negative. This negative component could be balanced by introduction of a force of interaction with the wall, representing, e.g., a short-ranged repulsive electrostatic potential. Since our simulations are restricted to two-dimensional motion, it is not necessary to introduce this force. However, this force would affect the flow field by contributing a vertical Stokeslet. Fig. S4 compares the flow field in the vicinity of a particle with and without the Stokeslet term. We find that the difference is quantitative, not qualitative.

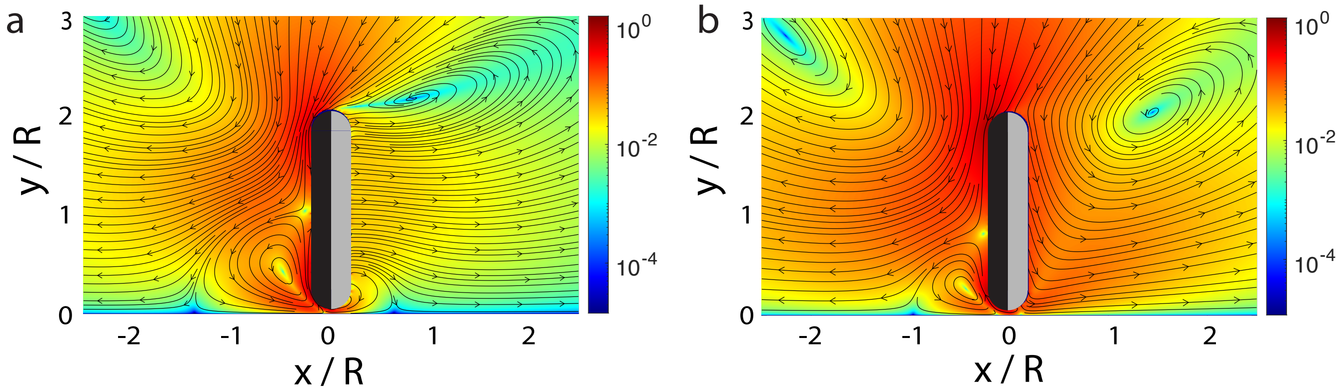

FIG. S4. **Flow field in the vicnity of Janus discoid.** (a) Side view of the flow field in the vicinity of a Janus discoid in the DC limit, from Fig. 6 in the main text. In calculating the flow field, we assumed the z-component of the particle's velocity is zero, which implicitly introduces an external force, and therefore a Stokeslet contribution to the flow field. (b) Flow field calculated for the same discoid as in (a), but with the z-component of the velocity non-zero, i.e., using the z-component obtained from the microscopic model detailed in the main text. Since the z-component is negative, streamlines issuing from the front of the particle are sloped downward. It may be observed that the flow field is quantitatively different from (a), e.g., it is faster in front of the particle, but has qualitatively the same structure.

V. BOUND STATE FORMATION IN THE POINT-PARTICLE MODEL

As described in the main text, the point-particle model can yield stable bound states for oblate or discoidal pusher-type squirmers. As an illustrative example, in Fig. S5 we show a pairing trajectory for two particles in a nearly head-on collision, obtained with the point-particle model.

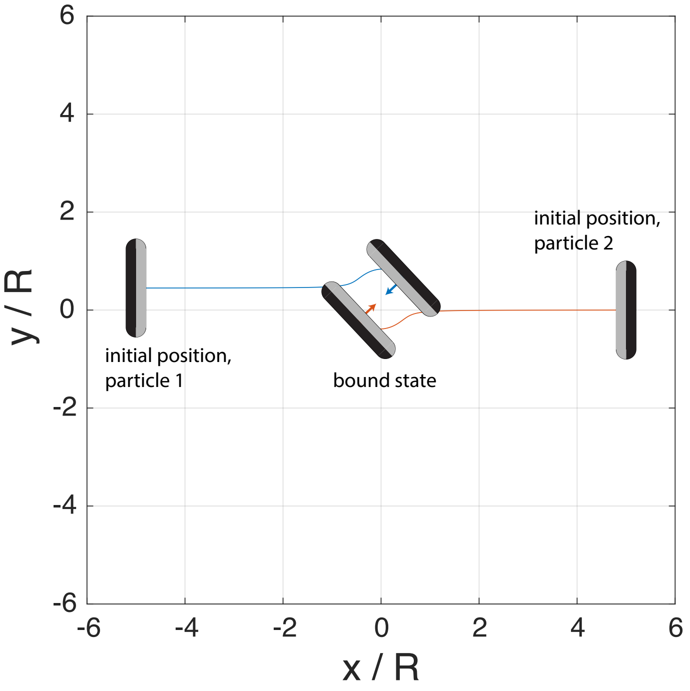

FIG. S5. **Pairing of two particles in the point-particle model.** For this example, we choose $\Gamma = -0.92$ and $\sigma_0/\mu R^2 = -2$, representing oblate or discoidal pushers. Even with initial offset of the particles' y-positions, the particles achieve the stable separation d predicted for two "pushers" in a head-on configuration.

VI. SUPPLEMENTARY MOVIES

Movie S1: **Reduced motility of active discoidal Janus ICEP particle system through pair formation.** 10 V_{pp}, 500 Hz, 30 fps. Playback: real-time.

Movie S2: **Visualization of flows behind a Janus discoidal particle using 1 μm tracer particles.** 10 V_{pp}, 500 Hz, 120 fps. Playback: 0.25x real-time.

Movie S3: **Collision of two Janus particles with a small off-center distance resulting in a pair formation.** 10 V_{pp}, 500 Hz, 30 fps. Playback: real-time.

Movie S4: **Collision of two Janus particles with a large off-center distance.** 10 V_{pp}, 500 Hz, 30 fps. Playback: real-time.

Movie S5: **Trapping of active particles on the metal sides of a formed pair configuration.** 10 V_{pp}, 500 Hz, 30 fps. Playback: real-time.

Movie S6: **Destruction of the arrested motility state through frequency switching.** $10V_{pp}$, 500 Hz to 250 kHz at t = 18s, 30 fps. Playback: real-time.
